# Associations between autistic traits, depression, social anxiety and social rejection in autistic and non-autistic adults

**DOI:** 10.1038/s41598-024-59532-3

**Published:** 2024-04-20

**Authors:** Emine Gurbuz, Deborah M. Riby, Mikle South, Mary Hanley

**Affiliations:** 1 Department of Psychology, University of Portsmouth, King Henry Building, King Henry I Street, Portsmouth, PO1 2DY UK; 2 Department of Psychology, Durham University, Durham, UK; 3 Emory University School of Medicine and Emory Brain Health Center, Atlanta, USA; 4 Centre for Interaction, Development and Diversity, University of Portsmouth, Portsmouth, UK; 5 Centre for Neurodiversity & Development, Durham University, Durham, UK

**Keywords:** Human behaviour, Neuroscience, Psychology

## Abstract

Autistic people frequently experience negative judgements from non-autistic people, often fuelled by misconceptions that autistic people lack empathy. Understanding responses to negative social judgement among autistic people is crucial because of the potential negative impact on wellbeing and future interactions. We investigated the role of autistic traits, social anxiety, and depression on behavioural indices of social rejection in 20 autistic (AUT; 11 males) and 40 non-autistic (N-AUT; 21 males) university students. Participants completed the Social Judgement Task (SJT) where they predicted whether they were liked by another person, then received feedback on whether those evaluations were correct. Participants also completed an Age Judgement Task (AJT) where they estimated the age of the pictured person. The AUT group had lower positive expectation scores, meaning less tendency to predict being liked. Across the whole sample, higher social anxiety predicted greater tendency to anticipate rejection from others, not autistic traits. These findings suggest early experiences of rejection might lead to a negative self-bias in autistic people and emphasise the importance of using a transdiagnostic approach by showing that social anxiety rather than autistic traits is associated with expectation of social rejection.

## Introduction

The capacity to perceive and evaluate social cues is a vital component of social interaction and can play a critical role in making judgements about the people we interact with including trustworthiness and approachability^1,2^. Neurotypical people make frequent judgments of the faces of social partners that guide social interactions, and equally those partners make judgements to guide their own behaviour – it is a two-way process^3^. Understanding how we respond to social judgements made by others is important for understanding real world social behaviours and experiences. When this judgement is positive, we experience social acceptance, but when this judgement is negative, we experience social rejection. Frequent feelings of social rejection can impact mental health, such as contributing to increased depression^4^ and anxiety^5^. Studies of social rejection in neurotypical adults using behavioural measures have shown that people are more likely to predict being liked by others even when others are unfamiliar to us^6–8^, suggesting that we like others to have a positive opinion of us and it makes us feel good.

Exploring whether social rejection experiences are the same or different for autistic adults is important for understanding broader social experiences of the autistic community. Many autistic young adults have reported experiencing loneliness, social isolation, peer rejection, bullying, stigma, and lack of social support networks^9–14^. These negative social experiences can create a risk for poor mental health. Indeed, frequent experiences of social rejection and loneliness^15^ contribute to high rates of depression and social anxiety in autistic individuals^16,17^. Given the moderating role of rejection sensitivity in developing depression and anxiety in neurotypical individuals^18^ and early life events of rejection leading to development of depression via changing the neurobiological responses^4^, it is crucial to investigate the associations between social rejection and mental health, particularly anxiety and depression. In addition, stigma and misconceptions about autistic people are very common among non-autistic community, which can also impact mental health of autistic people as well as social connectedness with others^19^. For example, due to the stereotypical assumption of reduced social interest, non-autistic individuals might have lower expectations of inclusion and social reciprocity in their interactions with autistic people^20^. In turn, this leads to higher expectations of negative judgements in autistic adults, including being rejected by others.

There is limited research on what factors interplay with experiences of social rejection, more specifically expectation of social rejection from others. Previous research suggested that adverse life events including rejection and bullying might explain higher mental health challenges in autistic people^21^. For example, autism acceptance from society predicted depression, but not anxiety in autistic adults^20^. Another study using a longitudinal design tested the interplay between autistic traits, self-compassion, and symptoms of anxiety and depression in autistic and non-autistic adults^22^. One of the components of self-compassion is self-kindness which means to treat oneself with kindness and support rather than being self-critical or self-judgemental^23^. The results showed that low self-compassion in autistic adults predicted later anxiety and depression, not the other way around. These studies suggested that poor mental health in autistic people might result from adverse life events including lack of acceptance from others, self-blame and negative self-judgement. However, it is not clear what autistic individuals think about how they are perceived by others (e.g. likeability) and what contributes to greater expectation of rejection (higher expectations of being unliked by others). Considering high co-occurrences of anxiety and depression together with experiences of social rejections in autistic individuals, it is important to understand the relationships between autistic traits, social anxiety or depression in both autistic and non-autistic individuals transdiagnostically, regardless of diagnosis.

One paradigm that has been used to empirically study the experience of social rejection is the *Social Judgement Task* (SJT)^24^. In the SJT, participants send their profile picture to the researcher and are told that the picture will be judged by a panel of unfamiliar adults. When the participants come to the lab, they are presented with pictures of other people who they are told have already made a judgement about them. The participant's task is then to make a judgment on whether this person liked them or not. This is followed by feedback indicating either social acceptance or social rejection by the other person. However, nobody has actually seen their profile picture and all feedback is randomly generated. To make sure that this response is specific to expectation of social rejection and not to general tendency to say “No”, participants also complete a control task where they judge the age of the same faces/people. Previous fMRI research using this paradigm found that adults show differential neural responses such that ventral anterior cingulate cortex (vACC) was sensitive to unexpected negative social feedback (social rejection) while dorsal anterior cingulate cortex (dACC) was sensitive to positive social feedback (social acceptance) and non-social negative feedback^24^.

Despite evidence showing that some autistic individuals can experience social rejection more frequently than their non-autistic peers, little is known about the relationship between expectation of rejection and mental health in autistic and non-autistic people. This is the first study to investigate behavioural indices of social rejection in autistic and non-autistic young adults using the SJT and the role of mental health and autistic traits in expectation of social rejection in a sample including people with high autistic traits.

### Study predictions

1. Non-autistic participants were expected to make comparatively more predictions of being liked while autistic participants were expected to make comparatively more predictions of being unliked/rejected.
2. We expected that higher autistic traits, social anxiety and depression symptoms would be associated with greater expectation of social rejection in all participants.

## Results

### Clinical and demographic variables

The AUT group (*M* = 34.65) had higher AQ scores than the N-AUT group (*M* = 16.46) (*t*(57) =  − 8.208, *p* < 0.001, *d* = 2.21) (See Table 1). The DASS-21 total scores and its subscales of *depression, anxiety*, and *stress* were calculated for each participant. The AUT group had significantly higher DASS-21 total scores compared to the N-AUT group, *t*(57) =  − 3.121, *p* = 0.004, *d* = 0.92. When each subscale was compared, the autistic students scored significantly higher on *anxiety, t*(57) =  − 3.058, *p* = 0.005, *d* = 0.90, and *stress, t*(57) =  − 4.035, *p* < 0.001, *d* = 1.04, but not on the *depression* subscale, *t*(57) =  − 1.603, *p* = 0.120, *d* = 0.47. Similarly, the AUT group reported significantly higher social anxiety scores measured with LSAS compared to N-AUT, *t*(56) =  − 6.254, *p* < 0.001, *d* = 1.90. Specifically, the LSAS subscales of *fear, t*(56) =  − 6.572, *p* < 0.001, *d* = 1.97, and *avoidance, t*(56) =  − 5.506, *p* < 0.001, *d* = 1.67, were significantly higher for autistic participants compared to their non-autistic peers.

**Table 1:** Clinical Scores of autistic traits, social anxiety, and depression in the AUT (N = 20 for DASS-21 and N = 19 for LSAS measures) and N-AUT group (N = 39).

		AUT Mean (SD) Range	N-AUT Mean (SD) Range	*p* value (group differences)
AQ Total		34.65 (8.74) 16–48	16.46 (7.69) 4–34	< 0.001
DASS-21 Total		42.20 (25.43) 10–108	22.95 (16.08) 2–82	0.004
	Anxiety subscale	11.70 (8.93) 0–36	5.08 (5.25) 0–18	0.005
	Depression subscale	11.60 (10.03) 0–32	7.59 (6.93) 0–36	0.12
	Stress subscale	18.90 (9.85) 6–42	10.05 (6.84) 0–28	< 0.001
LSAS Total		76.74 (26.05) 36–137	36.69 (14.36) 7–73	< 0.001
	Fear subscale	40.26 (12.77) 15–69	19.23 (8.04) 5–38	< 0.001
	Avoidance subscale	36.48 (13.98) 20–68	17.46 (7.99) 2–35	< 0.001

AQ: Autism Quotient. DASS-21: Depression Anxiety and Stress Scales- 21 Items. LSAS: Liebowitz Social Anxiety Scale.

### Behavioural results

#### Social judgement task

One-sample *t*-tests to check whether the expectation of rejection (negative expectation score) or acceptance (positive expectation score) differed significantly from chance (50%) found no expectation bias in either group (see Table 2; AUT; *t*(19) =  − 1.879, *p* = 0.90, *d* = 0.40, N-AUT; *t*(38) = 0.754, *p* = 0.456, *d* = 0.12). However, on average, the AUT group had lower positive expectation scores (lower tendency to predict being liked; group mean 44.25%) compared to the N-AUT group (group mean 51.45%) and this group difference was significant, *t*(57) = 2.038, *p* = 0.046, *d* = 0.54. The exact analysis with the responses from the AJT can be found in the Supplementary information (S1). This analysis found no differences between “Yes'' and “No” responses in AJT in the AUT group suggesting that the tendency to give more “No” responses in the SJT was task specific and cannot be explained by autistic participants having lesser tendency to answer affirmatively to yes/no questions.

**Table 2:** Behavioural results of social and age judgement tasks.

Task	Responses (SD)	AUT	N-AUT
AJT	Positive expectation score (% of ‘Yes’ judgements)	51.70 (9.32)	54.97 (10.36)
SJT	Positive expectation score (% of ‘Yes’ judgements)	44.25 (14.38)	51.45 (12.02)

#### Individual differences in experiences of social rejection

To test the hypothesis that higher autistic traits, social anxiety and depression symptoms would be associated with higher expectation of social rejection in all participants, one-tailed Pearson correlations were performed to check the linear relationships between behavioural responses from the SJT and autistic traits, social anxiety, and depression scores across participants. As can be seen in Table 3, negative expectation scores indicated by percentage of ‘No’ responses in the SJT were significantly correlated with autistic traits, *r* = 0.356, *p* = 0.006 (moderate positive correlation), social anxiety, *r* = 0.507, *p* < 0.001 (strong positive correlation), and depression scores, *r* = 0.374,* p* = 0.004 (moderate positive correlation). Participants with a higher negative expectation score (higher tendency to predict being rejected by others) were more likely to report higher autistic traits, higher social anxiety, and more depression symptoms.

**Table 3:** Correlations between experiences of social rejection and autistic traits, social anxiety, and depression symptoms across participants (N = 58).

Variables	1	2	3	4
1. Negative expectation score	1	0.356**	0.507**	0.374**
2. AQ total score		1	0.760**	0.379**
3. LSAS total score			1	0.471**
4. DASS-21 depression				1

AQ: Autism Quotient. LSAS: Liebowitz Social Anxiety Scale. DASS-21: Depression Anxiety and Stress Scales- 21 Items. ** Correlation is significant at the 0.01 level (1-tailed).

Hierarchical regression was conducted with AQ, DASS-21 depression scores and LSAS as predictors of negative expectation scores. Tests to see if the data met the assumption of collinearity indicated that multicollinearity was not a concern (VIF > 1.000). The data met the assumption of independent errors (Durbin-Watson value = 2.159). The histogram and P-P plot of standardised residuals indicated that the data contained normally distributed errors. The overall regression model was significant, *F*(3, 57) = 7.669, *p* < 0.001, *R*^*i*^ = 0.299. However, only social anxiety was a significant predictor, *β* = 0.408, *t*(57) = 2.212, *p* = 0.031. Thus, higher self-reported social anxiety significantly predicted higher tendency to expect being rejected by others. Adding depression symptoms and autistic traits did not improve the model as they were not significant predictors of negative expectation scores (DASS-21 depression subscale; *β* = 0.361, *t*(57) = 1.791, *p* = 0.079, AQ; *β* = -0.014, *t*(57) =  − 0.075, *p* = 0.941).

## Discussion

This study was the first to look at expectation of social rejection in autistic and non-autistic participants and its relation to autistic traits, social anxiety, and depression in a sample consisting of both autistic and non-autistic adults. The findings reveal some important new insights that are particularly important for understanding the experiences of autistic people. First, there was not a bias towards being liked than unliked by others in non-autistic participants, which was in contrast with previous findings^6,7,25,26^. However, when compared to autistic participants, non-autistic participants did predict being liked more, even though autistic participants did not make a strong prediction that others would unlike them. The lack of positive or negative self-evaluation bias in either group could be explained by individual differences in self-evaluation bias in the current sample, as discussed below.

Autistic participants expected to be liked less by others indicated by greater expectations of social rejection than non-autistic participants. These results imply that experiences of social rejection in autistic people might be quantitatively different than non-autistic individuals due to more common experiences of social rejection in autistic people. Given the prevalence of rejection and negative judgements outlined in the introduction, and that only 7% of autistic people feel accepted by society as an autistic person^20^, it is not surprising that autistic people anticipate more rejection, leading to potential increased social withdrawal and poorer mental health over time. Moreover, recent empirical evidence showed that non-autistic individuals made more negative judgements about autistic individuals^27,28^ and lower interactional rapport was reported between autistic and non-autistic adults^29^. Such research might explain why autistic people approach new situations thinking that they will be rejected again, due to many years of negative experience till adulthood. Longitudinal research is required to understand how negative childhood experiences including peer rejection, stigma, and stress influence future social experiences and interactions with others (e.g. friendships) in autistic people.

Next, individual differences in expectation of social rejection were investigated. Behavioural negative expectation scores (the negative self-evaluation bias) were found to be associated with higher self-reported autistic traits, social anxiety, and depression symptoms across participants. Subsequent regression analyses found that only social anxiety predicted higher expectations of rejection across participants, irrespective of autistic traits, suggesting that individuals who have higher social anxiety might be more prone to expect being rejected, irrespective of whether or not they have a diagnosis of autism. This relationship between social anxiety and negative self-evaluation bias has also been shown in individuals with Social Anxiety Disorders^30,31^. As discussed above, the negative self-evaluation bias among socially anxious individuals could be due to learned experiences from frequent social rejection in the past. According to the Compassionate Brain Theory, lower self-compassion including negative self-perception in individuals with high autistic traits might be a result of frequent negative experiences throughout one’s life^22^. Moreover, social anxiety and negative self-perception (e.g. “I am undesirable”) may lead to withdrawal from social interactions resulting in further isolation or depression^4,32^.

Social anxiety is not only observed autistic people, and therefore it should be taken into consideration in understanding transdiagnostic negative social experiences, particularly social rejection. However, given the higher levels of social anxiety in autistic people^33^ and apparent similarities in clinical presentations of psychiatric disorders in autistic and non-autistic people^34^, it is important to examine the relationship between social anxiety and social experiences of rejection and isolation in autistic populations as well as transdiagnostically. Another future direction for research is to compare a group of non-autistic participants matched on social anxiety to the autistic participants in order to understand whether the current patterns of associations are related to autistic traits or social anxiety per se.

There are several methodological considerations for the current study. The first methodological issue is what the social and age judgement tasks meant to autistic participants. For example, while the age judgement task was used intended as a control, non-social feedback condition, similarities in the task could lead autistic participants to find it equally social: all stimuli were still faces and both tasks were performed in front of an experimenter, meaning that responses to both tasks was provided essentially in a social situation. Future research may explore the validity of experimental paradigms for autistic people and not assume that tasks mean the same thing for autistic and non-autistic participants. The AUT sample was smaller than the N-AUT sample and autistic participants self-reported their autism diagnosis; larger, verified autism samples will provide more insight on individual and transdiagnostic group differences. The role of gender in responses to social rejection was not examined in the current study but is important for future research especially in the context of significant current interest in autistic females. That said, inclusion of non-binary genders, who might be more vulnerable to rejection and stigmatisation is necessary^35^^.^. Adding a state measure of social anxiety after the SJT could give more insight into experiences of immediate distress following social rejection in non-autistic and autistic participants and add understanding of individual differences and potential mechanisms underlying the experiences of social rejection. Information about co-occurring diagnosis for autistic people were obtained, however similar information was not collected for the non-autistic group, which is a limitation. Lastly, longitudinal data is needed to test whether social anxiety or expectation of rejection comes first, especially given the bidirectional relationship between the two as suggested in previous research^20,22^.

This was the first study to administer the Social Judgement Task to autistic young adults, using behavioural responses to probe experiences of social rejection. Autistic participants expected more rejection than their non-autistic peers, and social anxiety was a significant predictor of higher expectations to be rejected in all participants, irrespective of autism diagnosis. Social anxiety due to early negative experiences of rejection rather than difficulties associated with autism per se might explain why autistic people predict further rejection from others. Understanding the reasons behind higher expectations of social rejection in autistic individuals and whether it is associated with negative past experiences is needed to improve well-being of autistic individuals. Studying the long-term consequences of adverse childhood experiences, rejection, and stigma in autistic people is crucial to create an inclusive and welcoming society.

## Methods

### Participants

Forty non-autistic participants (N-AUT; 21 males, mean age = 22.83 years, SD = 4.13) and 20 autistic participants (AUT; 11 males, mean age = 23.58 years, SD = 4.27) participated in this study. Autistic participants were recruited via disability support services in the UK universities and they all had an official diagnosis of autism to be registered in support services. Non-autistic participants were recruited from UK universities via flyers distributed across the campus. Participants received £10 for their participation. The groups were matched in terms of chronological age, *t*(58) = 0.652, *p* = 0.52, and gender, x^2^ (1, *N* = 60) = 0.033, *p* = 0.86. Thirteen of the 20 autistic participants (65%) self-reported co-occurring diagnoses with eight (40%) experiencing co-occurring mental health issues. Autistic traits were measured using the Autism Quotient^36^.

### Ethics and inclusion statement

Ethical approval was provided by Durham University Psychology Department Ethics Sub-committee prior to the commencement of the study. All methods were carried out in accordance with relevant guidelines and regulations. Informed consent was obtained from all participants for study participation and publication of their data in an online open-access publication. All information obtained during the study are kept confidential and the data presented in the tables and figures does not include any identifiable information.

### Task descriptions and procedure

Adapted versions of Social and Age Judgement Tasks used by Gunther Moor, Crone and Van Der Molen^6^ were administered using E-prime 3.0 software^37^. Participants were told the task focused on forming first impressions among peers from multiple universities. First, they were asked to send a profile picture (e.g. a neutral expression head and shoulder shot) to the researcher approximately two weeks before the experiment. Participants were then told that their picture would be sent to a panel of students from other universities who would form first impression of them based on the picture. When participants arrived for the study, they were informed that they would first estimate the judgements of those who had already seen their profile picture and made a judgement whether they liked them or not (the SJT). Then, they were told that it was their turn to make judgments about the same students, and this judgement would be about the persons’ age (the AJT). Therefore, all participants completed the SJT first and then the AJT. Participants completed 4 practice trials followed by 121 trials in total for each task, with a break after the 60th trial. The feedback was randomly created by the computer programme such that there were 50% ‘Yes’ and 50% ‘No’ feedback trials in both tasks (see below for further explanation). Behavioural data of percentage of ‘Yes’ and ‘No’ responses given by the participants were collected throughout the two tasks. At the end of the experiment, participants were asked whether they believed in the cover story, and they all confirmed that they did.

In both tasks, the same pictures were presented. Instead of taking our own pictures as done in the original study, we used pictures of young adults taken from the Chicago Face Database^38^. The stimuli consisted of 121 face images (measuring 3.9 × 4.5 cm) presented on the computer screen. The age (inter-rater reliability of the ratings; α = 0.896) and attractiveness (α = 0.998) of the pictures were all rated, and the pictures standardized by the researchers who developed the database. The stimuli consist of 60 female and 60 male faces with a neutral expression on a white background (102 Caucasian, 4 African American, 8 Asian, and 6 Hispanic).

#### Social judgement task (SJT)

**Figure 1:**
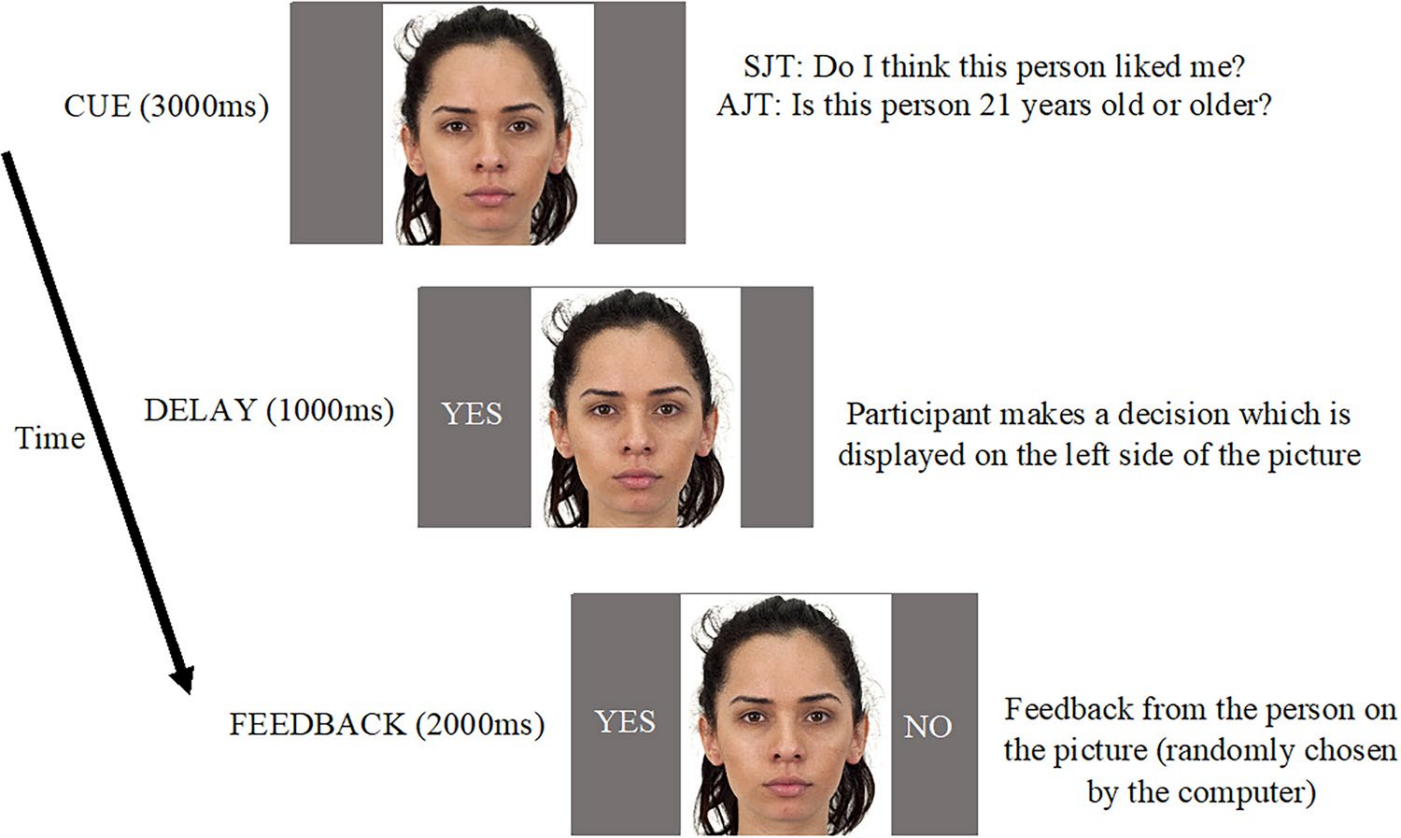
The timeline of SJT and AJT. *Note.* After a fixation point for 1000ms, the participant answers the question “Do I think this person liked me?” in SJT or “Is this person 21 years old or older?” in AJT. The response of the participant “YES” or “NO” is presented during the delay for 1000ms.

The timeline of the SJT paradigm can be found in Fig. 1. Each trial started with a fixation point for 1000 ms. Then the picture (cue) was presented for 3000 ms and it stayed on the screen until the end of the trial. During the presentation of the picture, the participants responded ‘YES’ by pressing on ‘1’ to indicate if they thought the person on the picture liked them or ‘NO’ by pressing ‘3’ to indicate if they thought the person on the picture did not like them. If the participants did not respond within 3000 ms, the feedback ‘too late’ was given. The participants’ response was then shown on the left side of the picture. After a delay of 1000 ms, the participants were provided with feedback on the supposed response from the person in the picture, with a ‘YES’ (the person on the picture liked them—*acceptance*) or ‘NO’ feedback (the person on the picture did not like them—*rejection*) presented on the left side of the picture. The feedback remained on the screen for 2000 ms. The next trial began with the fixation cross.

#### Age judgement task (AJT)

The AJT was very similar to SJT except for the type of judgement participants needed to make; the *age* of the person presented in the picture. The participants were presented with the same face stimuli for 3000 ms but this time they had to guess whether the person in the picture was 21 years old or older. The participants responded ‘YES’ by pressing on ‘1’ to indicate that the person on the picture was 21 years or older or ‘NO’ by pressing ‘3’ to indicate that the person on the picture was younger than 21 years old. 1000 ms after the participant’s response, the ‘YES’ (the person on the picture is 21 years or older) or ‘NO’ feedback (the person on the picture is not 21 years or older) was provided on the screen for 2000 ms (See Fig. 1).

### Self-report measures

Following the SJT and AJT, participants were asked to complete the Autism Quotient, Liebowitz Social Anxiety Scale, and Depression Anxiety Stress Scale-21 to measure their autistic traits, social anxiety, and depression respectively.

The Autism Quotient (AQ) is a 50-item self-report questionnaire to measure autistic traits in general population with IQ > 70^36^. The five subscales are social skill, attention switching, attention to detail, communication and imagination. Participants respond by choosing one of the four options for each item; ‘definitely agree’, ‘slightly agree’, ‘slightly disagree’, ‘definitely disagree’. Items are scored dichotomously (‘0’ or ‘1’) and the total scores range from 0 to 50, with higher scores indicating more autistic traits. In the present sample, the AQ demonstrated a high internal consistency (coefficient α = 0.930); AUT (α = 0.854); N-AUT (α = 0.872).

Liebowitz Social Anxiety Scale (LSAS) contains 24 items; 13 of which measure performance anxiety and 11 measure social interaction anxiety^39^. Participants rate each item for *fear* (0–3; none-severe) and *avoidance* (0–3; never-usually). In previous research, the self-report version of LSAS (LSAS-SR) has been reported to have good psychometric properties indicated by strong test–retest reliability (*α* = 0.83), internal consistency (*α* = 0.95), and convergent (*α* = 0.88 to 0.94) and discriminant validity^40^. When compared to the clinician-administered version (LSAS-CA), the LSAS-SR has been shown to be a valid measure with high internal consistency (*α* = 0.95) and identical subscale intercorrelations (*α* = 0.71–0.91)^41^ and it showed high internal consistency in autistic adults (*α* = 96)^42^. One participant in the AUT group did not complete the questionnaire. For the current sample with 19 participants in the AUT group and 40 participants in the N-AUT group, internal consistency of LSAS total scores was *α* = 0.959 (*avoidance* subscale; *α* = 0.918 and *fear* subscale; *α* = 0.929).

Depression Anxiety Stress Scale-21 (DASS-21)^43^ is a 21-item self-report measure of depression, anxiety and stress. On a 4-point Likert scale (from 1 = *did not apply to me at all* to 4 = *applied to me very much or most of the time*), participants rate each item based on its applicability to their life experiences over the past week, with higher scores indicating more severe symptoms. When administered to autistic adults, the DASS-21 has been reported to show good reliability and validity, with Cronbach’s alpha of 0.89 for the *depression* subscale, 0.83, for the *anxiety* subscale, and 0.86 for the *stress* subscale^44^. In the current sample, one N-AUT participant with a DASS-21 score of 112 (three SD above the mean) was excluded from further analysis. The internal consistency of DASS-21 total scores with 20 autistic and 39 N-AUT participants was *α* = 0.924 (*anxiety* subscale *α* = 0.834, *depression* subscale *α* = 0.793*, anxiety* subscale *α* = 0.836).

### Data analysis plan

Behavioural data consisted of the percentage of ‘Yes’ and ‘No’ responses given by the participants. In the SJT, if the participant made more than 50% of ‘Yes’ predictions, it would indicate a positive expectation score (that the participant expected to be liked) and if the participant made more than 50% of ‘No’ predictions, it would indicate a negative expectation score (that the participant expected not to be liked). One-sample *t*-tests were carried out with each group separately to check whether the expectation score differed significantly from 50%^45^. Group differences (AUT; N-AUT) in negative expectation scores were calculated by using independent samples *t*-test. Bonferroni correction was used for post hoc analyses and Huynh–Feldt corrections for violations of the assumptions of sphericity were used when necessary^46^.

Pearson bivariate correlations were calculated to check linearity between each predictor (AQ, LSAS, and DASS-21 depression scores) and the outcome variable (between negative expectation scores) across participants. After correction for multiple correlations and checking assumptions for multivariate regression, hierarchical regression analyses were performed to examine whether AQ, LSAS, and DASS-21 depression scores would predict behavioural responses to social rejection across participants. In the regression analysis, autistic traits from AQ were entered in Step 1, depression scores from DASS-21 were entered in Step 2 and social anxiety scores from LSAS were entered in Step 3. Analysis has been conducted using SPSS Statistics v27.

An a priori power analysis was conducted using G*Power version 3.1.9.7^47^ to determine the minimum sample size required to test the study hypothesis. Results indicated the required sample size to achieve 80% power for detecting a medium effect, at a significance criterion of α = 0.05, was *N* = 55 for linear multiple regression. Thus, the obtained sample size of *N* = 58 is adequate to test the hypothesis.

### Supplementary Information

Supplementary Information.

## Data Availability

All data generated and/or analyzed during this study are available from the corresponding author (E.G.) on request.
