# Supplementary material for: Associations between autistic traits, depression, social anxiety and social rejection in autistic and non-autistic adults
Source: Sci Rep. 2024 Apr 20;14:9065. doi: 10.1038/s41598-024-59532-3 (PMC11032319; doi:10.1038/s41598-024-59532-3)
Supplement: Supplementary file 1 — Supplementary Information. [file 41598_2024_59532_MOESM1_ESM.pdf]

Age judgement task

In the N-AUT group, the percentage of 'Yes' responses was significantly higher compared to a chance level of 50%, $t(38) = 2.995$, $p = .005$, $d = 0.48$; in contrast, participants in the AUT group did not show any bias in the AJT, $t(19) = .818$, $p = .423$, $d = 0.18$. These results suggested that non-autistic participants estimated the people in the pictures to be older than the autistic participants. In order to statistically test group differences in tendencies to say "Yes" or "No" in the AJT, same analysis as the SJT was conducted. No group differences were found in either responses of "Yes" ($t(57) = 1.184$, $p = .241$, $d = 0.33$) or "No" ($t(57) = -1.177$, $p = .207$, $d = 0.36$) in the AJT, indicating that the autistic participant did not have a general tendency to say "No".
